# Relative strength explains the differences in multi-joint rapid force production between sexes

**DOI:** 10.1371/journal.pone.0296877

**Published:** 2024-02-15

**Authors:** Paul Comfort, John J. McMahon, Jason P. Lake, Nicholas J. Ripley, N. Travis Triplett, G. Gregory Haff

**Affiliations:** 1 Directorate of Psychology and Sport, University of Salford, Salford, Greater Manchester, United Kingdom; 2 Strength and Power Research Group, School of Medical and Health Sciences, Edith Cowan University, Joondalup, Australia; 3 Chichester Institute of Sport, Nursing, and Allied Health, University of Chichester, Chichester, West Sussex, United Kingdom; 4 Department of Public Health and Exercise Science, Appalachian State University, Boone, North Carolina, United States of America; Sheffield Hallam University, UNITED KINGDOM

## Abstract

The primary aim of this study was to determine whether relative strength explains the differences in the rapid force production (force developed during first 150-, 200-, and 250 ms) of females and males, and to evaluate the relationships between peak force and rapid force production. Sixty-three team sport athletes (females: n = 25, age = 21.5 ± 1.3 years, stature = 166 ± 5 cm, body mass = 60.65 ± 10.04 kg; males: n = 38, age = 21.9 ± 1.1 years, stature = 178 ± 7 cm, body mass = 76.55 ± 12.88 kg) performed a series of isometric mid-thigh pull (IMTP) trials, with all participants’ data used for correlational analysis. After testing, females and males were divided into 20 strength-matched pairs, based on their relative peak force (peak force ∙ body mass). There were no meaningful differences between sexes for relative force at 150 ms (*g* = 0.007 [95% CI -0.627, 0.648]), 200 ms (*g* = -0.059 [95% CI -0.695, 0.588]) and 250 ms (*g* = -0.156 [95% CI -0.778, 0.473]). Similarly, when expressed as a percentage of peak force there were no meaningful differences in force at 150 ms (g = -0.015 [95.0%CI -0.650, 0.680]), 200 ms (g = -0.099 [95.0%CI -0.714, 0.559]) or 250 ms (g = -0.272 [95.0%CI -0.856, 0.328]) between strength-matched females and males. Based on the correlations, there were very large to nearly perfect relationships (r = 0.77–0.94, *p* <0.001) between peak force and rapid force production, with peak force explaining 59%, 77% and 89% of the variance in force at 150-, 200- and 250 ms, respectively. When comparing females and males, relative strength (based on body weight or a percentage of peak force) should be considered, and practitioners should be aware of the role of peak force in rapid force production.

## Introduction

There are clear differences in the athletic performances between females and males, whereby males regularly outperform females; as a result, competitions are normally separated based on sex. These performance differences are commonly reported as sex differences and it is postulated that these are underpinned by differences in genetics [1] and sex hormone concentrations [2]. Such theories may result from the fact that minimal differences in performances are observed between females and males before the onset of puberty [2, 3], and due to the greater rate of decline in performance in females during the perimenopausal period, compared to males of a similar age [3]. The differences observed during puberty are thought to be attributed to the differences in hormone concentrations between the sexes, with the higher androgen levels resulting in a more rapid increase in muscle mass in males from puberty onwards [2, 3], with greater muscle mass in adult males compared to females normally observed across the rest of the lifespan [4]. In addition, there are differences in the distribution of muscle mass, with females having more of their total muscle mass in the lower body [3]. This may explain, in part, the greater differences in upper body strength in males and females when compared to differences in lower body strength [5], even when strength is expressed relative to body mass.

When matched using relative strength (i.e., strength ∙ body mass) however, the differences between sexes are reduced substantially, sometimes completely [6, 7]. It should be noted that strength and the ability to generate high magnitudes of force over short epochs are key determinants of performance in athletic tasks, especially where one’s body mass has to be accelerated or decelerated [8, 9], because force relative to mass determines acceleration and impulse (force x time) relative to mass determines movement velocity. While genetics and hormonal function play a role in the rate of strength development, force production characteristics and rapid force production are clearly trainable [10–13], with maximum strength during multi-joint tasks being closely related to performance in numerous athletic tasks [14, 15]. More importantly, when force production characteristics are increased through appropriate training, performance in athletic tasks improves [16–18].

Staron et al. [19] demonstrated that early adaptations to resistance training, in individuals with no heavy resistance training experience, were comparable between young adult females and males, and strength was equal when expressed relative to fat free mass. Similar adaptations to early strength training in females and males are likely due to neurological adaptations and fibre type shifts, being the predominant adaptation, rather than increases in muscle mass [10–12, 19]. Adaptations to long-term strength training include neurological and architectural changes in addition to increases in muscle mass [20, 21], with the rate of change in muscle mass likely affected by differences in hormone production and the resultant differences in hormone concentrations between the sexes. In fact, in healthy individuals lower-body muscle mass in males is reported to be ~33% greater than females [4], which would likely result in increased absolute force production in males, highlighting the importance of scaling strength by body mass to account for differences in stature.

To date only Nimphius et al. [6] have compared absolute and ratio scaled (peak force ∙ body mass) peak force production of females and males during a multi-joint task (i.e., isometric squat), demonstrating that there are no sex-based differences in ratio scaled force production between strength-matched pairs, highlighting that performance differences may actually be strength-related differences and not sex differences. However, while maximal force production is associated with rapid force production [22, 23], it is generally the latter that is required to accelerate the athlete, or an object, during athletic tasks, due to the time-constrained nature of most sporting tasks. For example during high velocity sprinting, foot contact times can be much less than 250 ms, with a progressive decline in contact time as running velocity increases [24]. As such, the assessment of rapid multi-joint force production between strength-matched females and males should be evaluated to determine if relative strength explains any observed differences in rapid force production between the sexes.

The primary aim of this study was to compare rapid force production (i.e., force at 50-, 100-, 150-, 200- and 250 ms) and maximum multi-joint isometric force production between female and male athletes to determine if differences in relative strength (i.e., scaled by body mass and relative to peak force) account for the commonly observed differences in rapid force production between the sexes. A secondary aim was to determine if higher forces at early time points are related to peak force. It was assumed that males would demonstrate greater rapid force production at all time-points and greater peak force compared to females, but that these differences would be substantially reduced when forces were ratio scaled (peak force ∙ body mass), or scaled as a percentage of peak force. We also assumed that there would be a meaningful association between peak force and force at early time-points (i.e., force at 50-, 100-, 150-, 200- and 250 ms) and that the magnitude of this association would increase as time-points progress (e.g., a stronger association between peak force and force at 250 ms compared to 150 ms). It is expected that the results of this study will inform researchers interested in comparing females and males regarding appropriate scaling of data to ensure fair comparisons are made and help inform practitioners regarding training priorities for females.

## Materials and methods

An observational comparative research design was used to determine the differences in early (i.e., 50-, 100-, 150-, 200- and 250 ms) and maximum multi-joint isometric force production, assessed using the isometric mid-thigh pull (IMTP), between female and male athletes. Forces were subsequently ratio scaled to determine if body mass accounts for the higher force values regularly demonstrated by males, in line with Nimphius et al. [6] who demonstrated that differences between sexes may be explained my relative strength. Body mass rather than fat free mass was used for ratio scaling, as during sporting activities athletes must accelerate their entire mass and not only their fat free mass [25]. Force at each specified time-point was also expressed as a percentage of peak force, in line with previous research [22, 23].

### Participants

An *a priori* statistical power analysis (G*Power 3.1 [26]), based on the results of previous research [6] demonstrated that 28 participants (14 strength-matched pairs) were required for a β ≥ 0.95, with a moderate effect (*d* = 0.76) at an α ≤0.05. A total of 63 collegiate and semi-professional athletes (females: n = 25, age = 21.5 ± 1.3 years, stature = 166 ± 5 cm, body mass = 60.65 ± 10.04 kg; males: n = 38, age = 21.9 ± 1.1 years, stature = 178 ± 7 cm, body mass = 76.55 ± 12.88 kg), from a variety of team sports (Rugby League, Rugby Union, Soccer, Field Hockey) volunteered to participate in this investigation. All athletes had ≥2 years of structured resistance training experience. Participants had just completed a 4-week strength training meso-cycle at the time of data collection and were familiar with all testing procedures as the IMTP was regularly used to monitor their changes in force production capability. While there were some between participant differences in the exercises used in the preceding 4-week strength training mesocycle, all exercises had been performed using 3–5 sets of 3–5 repetitions at a relative intensity of 80–90% of one repetition maximum (1RM) for the primary exercises. All participants provided written informed consent to participate in the investigation, which was granted ethical approval by the institutional review board (HST2021-113), with all data stored in an anonymised format. Data was collected between February 2021 and July 2021, in a laboratory setting with a constant temperature and humidity. The study was conducted according to the 7^th^ revision of the Declaration of Helsinki.

Phase of the menstrual cycle was not considered as this has been shown to have a trivial effect on force production characteristics [27–29]. It was deemed important to collect data at a comparable point at the end of a training phase to ensure that the participants were in a comparable physiological state, without differences in fatigue associated with different training volumes across a phase of training.

### Procedures

Upon arrival at the laboratory each participant provided written informed consent and subsequently had their stature and body mass measured and recorded. Each participant then performed the normal dynamic warm-up that they would complete before their gym-based training, followed by a specific warm up of three sets of three repetitions of a dynamic mid-thigh pull, with ~50% of their self-reported 1RM power clean.

### Isometric mid-thigh pull assessment

The IMTP assessments were conducted in line with recommended procedures [30]. An immovable cold rolled steel bar was positioned at a height that replicated the start of the second pull phase of the clean on a custom rack (Absolute Performance, Cardiff, UK) above a force platform (type 9286AA, Kistler Instruments, Winterthur, Switzerland), interfaced with a laptop computer and specialist software (Bioware 3.1, Kistler Instruments, Winterthur, Switzerland), sampling at 1000 Hz. Once bar height was established, participants stood on the force platform with their hands strapped to the bar, using standard lifting straps. Each participants adopted a posture which replicated the start of the second pull phase of the clean, resulting in knee and hip angles of 138.5 ± 3.6˚ and 145.3 ± 2.9˚ respectively, in line with previous recommendations [30].

After the dynamic warm-up, each participant performed three warm-up trials; one at 50%, one at 75% and one at 90% of their perceived maximum effort, each separated by one minute of rest. Once body position was stabilized (verified by watching the participant and the force-time record), the participants were given a countdown of “3, 2, 1, Push”. Any obvious pre-tension, determined as a force >50 N above the participants’ body mass, was not permitted before the initiation of the pull, with feedback regarding this provided throughout the warm-up trials and the maximal effort trials. Participants were instructed to “push their feet into the ground as fast and hard as possible”, in line with previous recommendation [30, 31]. Each IMTP trial was performed for ~five seconds, after at least one second of quiet standing in position before the start of the pull [30]. Participants were provided with strong verbal encouragement during each trial. Each participant performed three maximal IMTP trials interspersed with two minutes of rest between trials. If peak force during all trials did not fall within 250 N of the best trial (e.g., if peak force was >250 N below the peak force during the best trial), the trial was discounted and repeated after a further two minutes of rest, in line with previous recommendations [30]. All participants performed three acceptable trials within a maximum of five maximal effort attempts.

### Data analysis

Raw unfiltered, force-time data were exported for subsequent analysis in to a bespoke Excel spreadsheet (Microsoft, Redmond, WA). The onset of force production was defined as an increase in force that was greater than five standard deviations of the mean force calculated during the last 1 second immediately before any clear and consistent increase in force [30, 32]. Body weight (the 1 second’s mean force) was subtracted from the force-time curve to provide the net force-time curve (gross force–body mass) to enable fair comparisons between females and males of differing body mass and to prevent inflation of the associations between peak force and time-specific variables which would occur if gross force was used, in line with previous research [22]. The peak force was reported as the maximum force across the recorded net force-time curve. Subsequently, force at 50-, 100-, 150-, 200- and 250 ms was identified (from the start point explained above). The mean of the three trials was used for further analysis. All force data were presented in absolute terms (newtons) and ratio scaled (e.g. peak force∙body mass, N∙kg) to provide fair comparisons between sexes. Absolute force at each time-point was also expressed relative to peak force (e.g., [force at 150 ms∙peak force] x 100) [22, 23].

To ensure a fair comparison of rapid force production between sexes, females and males were matched based on their relative peak force (peak force ∙ body mass), as closely as possible (<10% difference between pairs in line with previous research [6]), resulting in some participants (5 females and 18 males) being excluded from this part of the study if there were no comparable female or male participants (e.g., some males demonstrated higher relative peak force than any of the females and some females demonstrated relative peak force values lower than any of the males). After data analysis participants were divided into relative strength-matched groups (<10% difference between matched pairs), resulting in 20 females (age = 21.3 ± 1.1 years, stature = 163 ± 6 cm, body mass = 60.20 ± 10.61 kg, IMTP peak force = 25.37 ± 5.46 N^.^kg^-1^) and 20 males (age = 21.8 ± 0.8 years, stature = 179 ± 4 cm, body mass = 77.24 ± 13.46 kg, IMTP peak force = 25.39 ± 6.02 N^.^kg^-1^). There were no meaningful differences (*p* = 0.993, *g* = 0.003 [95%CI = -0.626–0.607]) between group IMTP relative peak force.

### Statistical analyses

Normality of all data was determined via Shapiro-Wilk’s test, with all variables normally distributed (*p* > 0.05). Relative reliability was assessed using two-way mixed model intraclass correlation coefficients (ICC: model 3,1) and 95% CI [33]. The ICC values were interpreted based on the lower bound 95% CI as poor (<0.50), moderate (0.50–0.74), high (0.75–0.90) and excellent (>0.90) [33]. Absolute reliability was determined by calculating the percentage coefficient of variation (%CV) and associated 95%CI, with <10% considered acceptable [34].

Independent t-tests and Hedge’s *g* effect sizes with Gardner-Altman estimation plots [35] were calculated to determine if there were any significant or meaningful differences, respectively, between females and males. The *a priori* alpha level was set at *p* < 0.05 and effect sizes classified as trivial (≤0.19), small (0.20–0.59), moderate (0.60–1.19), large (1.20–1.99), and very large (≥2.00) [36].

Pearson’s correlation coefficients, with 95%CI, and coefficient of determination (R^2^), were calculated, after a Fisher z transformation [37], to determine associations between peak force and force at 150-, 200- and 250 ms (force at 50 ms and 100 ms were excluded due to unacceptable reliability). The associated *p* values were adjusted using Bonferroni post-hoc correction for multiple comparisons, and correlations interpreted as <0.10, 0.10–0.29 0.30–0.49, 0.50–0.69, 0.70–0.89 and ≥0.90 as trivial, small, moderate, large, very large and nearly perfect, respectively [36]. Statistical analyses were conducted in SPSS (version 25, IBM), www.estimationstats.com/#/ [38] and JAMOVI (Version 1.2.27).

## Results

### Reliability

For both sexes, force at 50 ms demonstrated moderate relative reliability, but poor absolute reliability, while relative reliability of force at 100 ms was poor for females and high for males, and absolute reliability was not acceptable for either (Table 1). Force at 50 ms and 100 ms were therefore excluded from further analysis. In contrast, relative reliability for peak force and force at 150-, 200- and 250 ms was high, with acceptable absolute reliability (%CV <10%) for both sexes (Table 1).

**Table 1:** Descriptive characteristics and reliability statistics for absolute net force variables.

Variable	Sex	Mean (SD)	ICC (95%CI)	%CV (95%CI)
Force at 50 ms (N)	Female	293.5 (25.6)	0.833 (0.743–0.957)	*25*.*6 (22*.*6–28*.*7)*
	Male	423.4 (181.1)	0.815 (0.646–0.919)	*17*.*7 (14*.*8–20*.*6)*
Force at 100 ms (N)	Female	547.9 (139.4)	*0*.*729 (0*.*481–0*.*892)*	*10*.*6 (7*.*8–13*.*4)*
	Male	725.7 (292.6)	0.902 (0.801–0.958)	*12*.*1 (9*.*3–14*.*9)*
Force at 150 ms (N)	Female	821.9 (164.0)	0.917 (0.800–0.971)	8.6 (5.8–11.4)
	Male	1084.9 (407.7)	0.975 (0.945–0.990)	9.4 (6.6–12.2)
Force at 200 ms (N)	Female	1014.8 (199.5)	0.952 (0.883–0.983)	6.8 (4.0–9.6)
	Male	1309.8 (397.4)	0.969 (0.933–0.988)	8.4 (5.6–11.2)
Force at 250 ms (N)	Female	1096.6 (241.5)	0.974 (0.938–0.991)	5.9 (3.1–8.6)
	Male	1386.8 (381.8)	0.967 (0.938–0.987)	8.2 (5.4–11.0)
Peak Force (N)	Female	2100.0 (431.3)	0.996 (0.990–0.999)	2.8 (0.1–5.6)
	Male	2732.6 (734.2)	0.990 (0.979–0.996)	3.6 (0.8–6.3)

SD = standard deviation; ICC = intraclass correlation coefficient; CI = confidence interval; CV = coefficient of variation; *values in italics highlight that values exceed the acceptable limits for reliability*

### Absolute force

**Fig 1:**
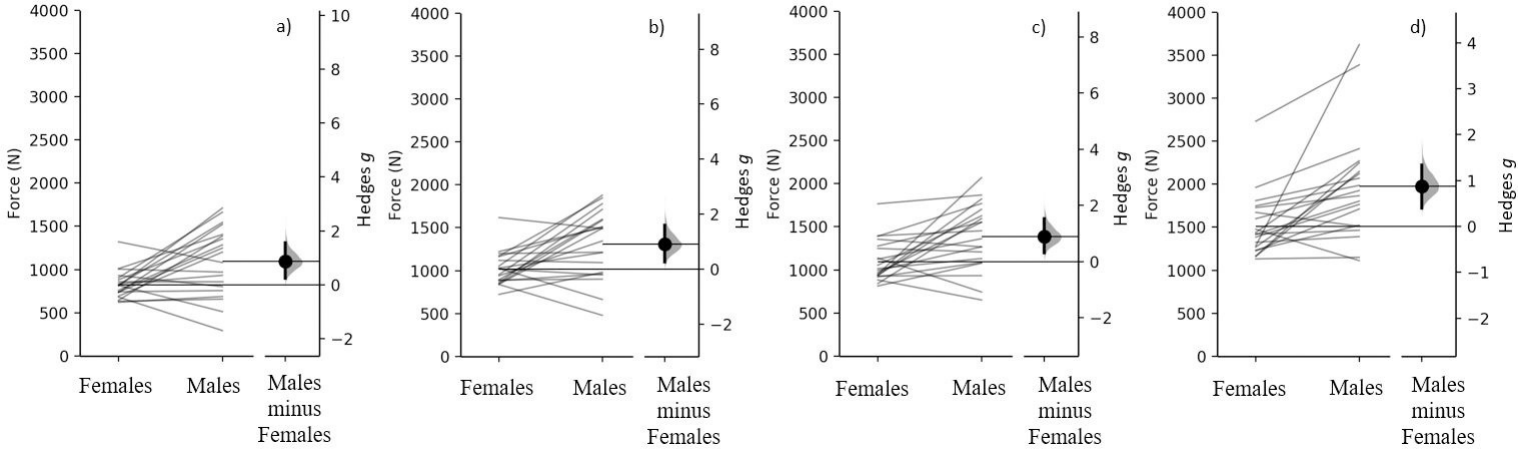
Comparison of absolute net force at a) 150 ms, b) 200 ms, c) 250 ms and d) absolute peak force between females and males. Paired females and males are plotted on the left axes, with each paired set of observations connected by a line. The paired Hedges *g* effect size and 95% confidence interval is plotted on the right axes, as Gardner-Altman estimation plots.

Males produced a moderately greater absolute net force at 150 ms (*g* = 0.87 [95%CI 0.16, 1.56], p = 0.007), 200 ms (*g* = 0.92 [95%CI 0.17, 1.65], p = 0.006) 250 ms (*g* = 0.89 [95%CI 0.17, 1.6], p = 0.006) and absolute net peak force (*g* = 0.87 [95%CI 0.16, 1.38], p = 0.005) compared to females (Fig 1, Table 1). It should be noted that, on an individual basis, some females demonstrated greater absolute time-related forces and absolute peak force compared to strength-matched males (Fig 1).

### Relative force

There were no meaningful differences between sexes for relative force at 150 ms (females = 14.01 ± 3.44 N^.^kg^-1^ vs. males = 14.05 ± 5.29 N^.^kg^-1^, *g* = 0.007 [95% CI -0.63, 0.65], *p* = 0.981), 200 ms (females = 17.17 ± 3.51 N^.^kg^-1^ vs. males = 16.92 ± 4.73 N^.^kg^-1^, *g* = -0.059 [95% CI -0.70, 0.59], *p* = 0.850) and 250 ms (females = 18.46 ± 3.65 N.kg^-1^ vs. males = 17.87 ± 3.81 N^.^kg^-1^, *g* = -0.156 [95% CI -0.78, 0.47], *p* = 0.624). Similarly, there were no meaningful differences in relative peak force between sexes (females 25.37 ± 5.46 N^.^kg^-1^ vs. males, 25.39 ± 6.02 N^.^kg^-1^, *p* = 0.993, *g* = 0.003 [95%CI = -0.630–0.610]).

When expressed as a percentage of peak force there were no meaningful differences in force at 150 ms (females = 55.5±8.8%, males = 55.3±16.2%; p = 0.996, g = -0.015 [95.0%CI -0.65, 0.68]), 200 ms (females = 68.1±6.9%, males = 67.0±13.0%; p = 0.759, g = -0.099 [95.0%CI -0.71, 0.56]) or 250 ms (females = 73.1±5.8%, males = 71.0±8.0%; p = 0.373, g = -0.272 [95.0%CI -0.86, 0.33]), between strength-matched females and males.

**Fig 2:**
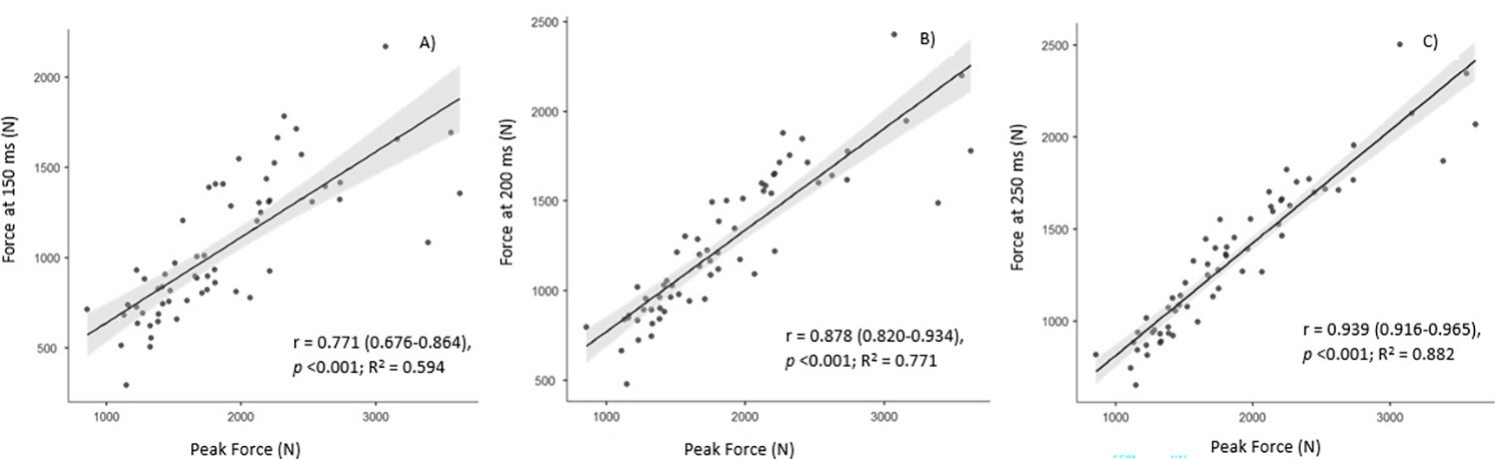
Correlations between absolute net peak force and absolute net force at a) 150 ms, b) 200 ms and c) 250 ms, for all participants (n = 63). The shaded area represents the 95% confidence intervals for the Pearson’s correlation coefficients.

Peak force demonstrated very large to nearly perfect associations (r = 0.771–0.939, *p* <0.001) with rapid force production (Fig 2).

## Discussion

The primary aim of this study was to compare rapid force production (i.e., force at 150-, 200- and 250 ms) and maximum multi-joint isometric force production between female and male athletes to determine if differences in relative strength account for the commonly observed differences in rapid force production between the sexes. A secondary aim was to determine if higher forces at early time points are related to peak force. This is the first study to demonstrate that strength matching females and males, eliminates the ‘sex differences’ in rapid force development (whether expressed relative to body mass or relative to peak force) during the IMTP, based on group means, highlighting the importance of developing relative strength in athletes, but especially in female athletes. In addition, there is a strong correlation between peak force and rapid force development with stronger correlations between peak force and rapid force development as the time point progresses through the task. These findings illustrate the importance of training individual athletes based on their relative strength capacity and not their sex.

**Fig 3:**
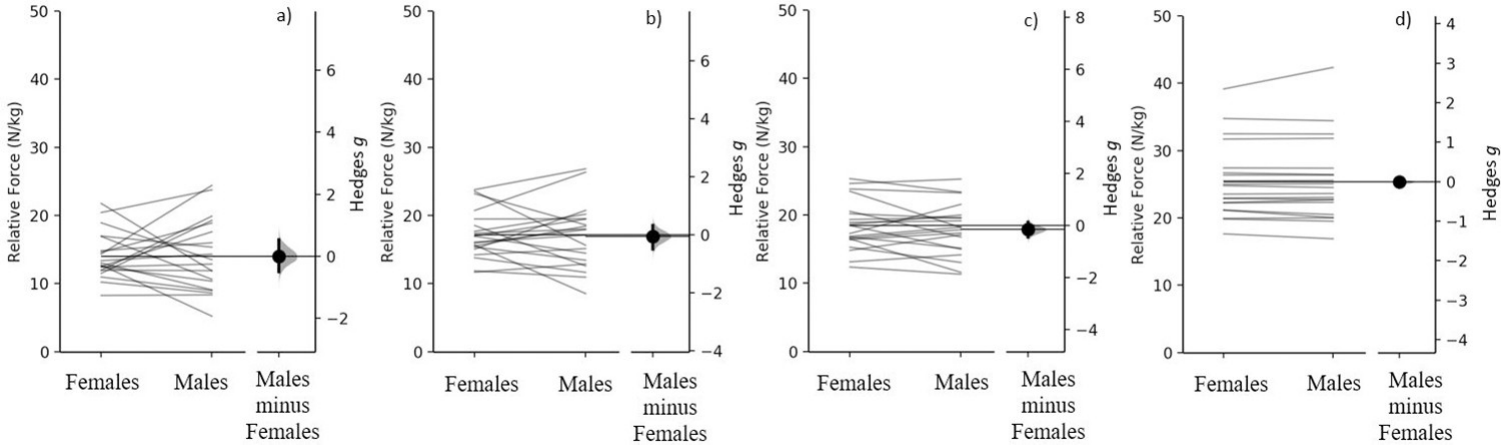
Comparison of relative net force at a) 150 ms, b) 200 ms, c) 250 ms and d) relative peak force between females and males. Paired females and males are plotted on the left axes, with each paired set of observations connected by a line. The paired Hedges *g* effect size and 95% confidence interval is plotted on the right axes, as Gardner-Altman estimation plots.

**Fig 4:**
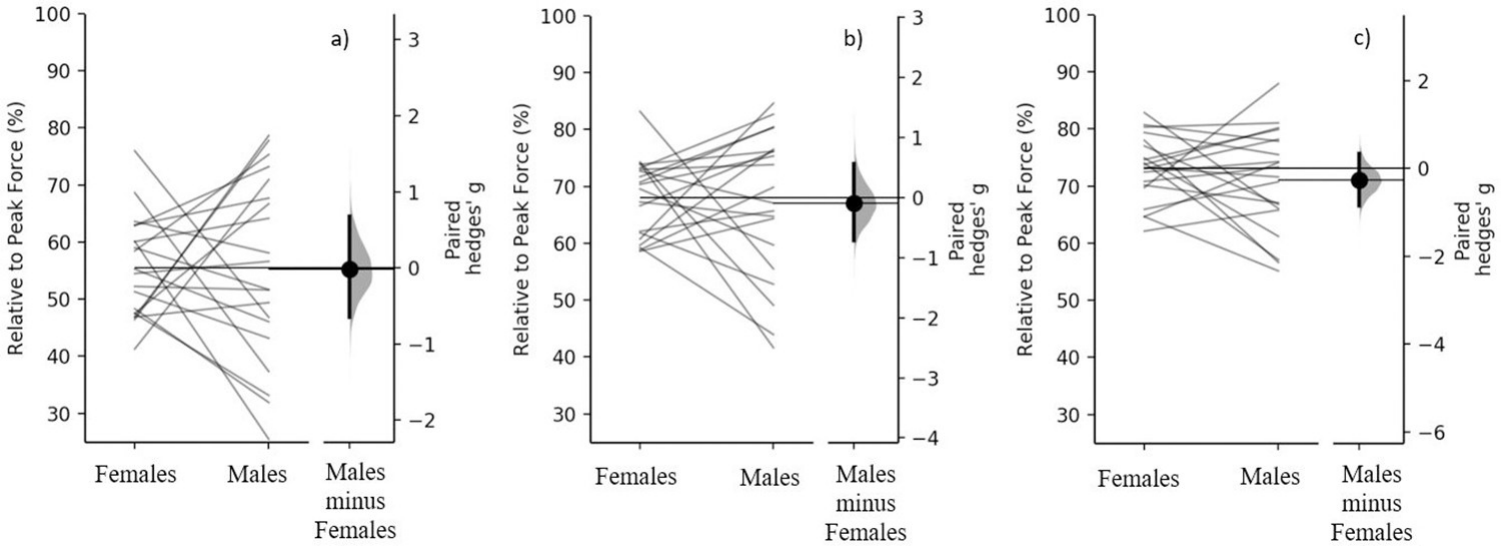
Comparison of force production at specific time points, a) 150 ms, b) 200 ms, c) 250 ms, expressed as a percentage of peak force, between females and males. Paired females and males are plotted on the left axes, with each paired set of observations connected by a line. The paired Hedges *g* effect size and 95% confidence interval is plotted on the right axes, as Gardner-Altman estimation plots.

Similar to the findings of Nimphius et al. [6], when strength-matched, based on relative net force (so that both body mass and strength are removed as confounding variables), differences in peak force production commonly referred to as sex differences are clearly strength differences. Based on our data moderately (*g* = 0.87 to 0.92) greater absolute peak force (34.1%) and forces at 150- (35.5%), 200- (31.5%) and 250 ms (29.9%) were demonstrated when males were compared to females (Fig 1). In contrast, when force was ratio scaled (divided by body mass) the differences in peak force (0.3%) and force at 150- (2.4%), 200- (-0.9%) and 250 ms (-2.7%) are almost completely removed, resulting in small to trivial (*g* = -0.156 to 0.003 [Fig 3]) differences, in line with the previous findings for peak force [6]. Similarly, when rapid force production was expressed as a percentage of peak force there were no meaningful differences (*g* = -0.02 to -0.27 between sexes [Fig 4]). When examining the individual differences in absolute and relative force at 150-, 200- and 250 ms, some females generated higher forces than the strength-matched males (Figs 1 and 3); however, further research is required to determine if these individual differences are because of differences in muscle architecture or fibre type distribution.

The results of this study and those presented by Nimphius et al. [6] highlight the importance of considering relative strength when making comparisons between sexes, as it appears that the differences in force production characteristics commonly reported between the sexes may be explained by differences in relative strength, as previously suggested [7]. Additionally, Nimphius [38, 39] also suggests that it may be the relative strength differences between the sexes that lead to a higher risk and incidence of injury. Because strength is a trainable quality, these findings also highlight the importance of appropriately planned strength training, especially for female athletes.

As early adaptations to strength training result in predominantly neurological adaptations and fibre type shifts, rather than increases in muscle mass [10–12, 19], initial changes in strength training have been reported to be similar between sexes [19]. Adaptations to long-term strength training include neurological and architectural changes in addition to increases in muscle mass [20, 21, 40], and while the rate of change in muscle mass is likely affected by differences in hormone concentrations between the sexes [41], hypertrophy and fibre type shifts (e.g., decreased type IIX and increased type IIA) have been demonstrated with heavy resistance training in females [42]. Such adaptations may explain why strength-matched pairs demonstrate similar rapid force production characteristics, due to the likely similar muscular adaptations, although additional research comparing fibre types and muscle architecture are required to confirm this. It is also worth noting that a greater muscle fibre cross sectional area has been observed in males, compared to females [43] and therefore a greater focus on hypertrophic adaptations, to provide an appropriate foundation for force production should be considered an important part of the periodized training of females athletes, to enhance performance and potentially reduce injury risk [6, 39].

In line with previous observations from single-joint assessment [44, 45], and the assessment of force production via the IMTP in weightlifters [46] and team sport athletes [22], peak force explains a large percentage (59%, 77%, 88%) of the variance in the force produced at 150-, 200- and 250 ms, respectively, with the magnitude increasing and the 95% CI narrowing as time point progresses from early to late epochs (150–250 ms). Such findings illustrate the importance of developing maximal force production to facilitate and enhance rapid force production, as previously recommended [8, 22].

The poor absolute reliability of force at 50 ms and 100 ms is a limitation of this investigation, but in line with lower levels of reliability commonly reported for force at earlier time-points compared to force at later time-points in the IMTP [13, 22] and during single joint assessments [47]. We suggest that researchers investigate the effects of strength on isometric force-time characteristics between the sexes at time-periods <150 ms and to further determine the effect of increased strength on the force-time characteristics between the sexes. If time permits, practitioners and researchers should also consider implementing one second ‘explosive’ IMTP trials to evaluate rapid force production in addition to the ‘traditional’ IMTP protocol to determine peak force, as the one second ‘explosive’ efforts are reported to improve reliability and increase the force generated at early time-points [48, 49] and in line with recommendations by Maffiuletti et al. [50]. Furthermore, researchers should also consider determining if differences in force-time characteristics are explained by differences in muscle architecture and fibre type and the adaptations which result from progressive and long-term strength training. Finally, the use of ratio scaling usually assumes an ‘almost perfect’ linear relationship between body mass and performance, while the correlation between body mass and strength for our data was strong (*r* = 0.74) it was not ‘almost perfect’. However, in most sporting activities athletes have to accelerate and decelerate their entire mass, making scaling by body mass very important.

## Conclusion

An athlete’s ability to express force is a key determinant in athletic performance; moreover, as most athletic tasks or sporting demands are constrained by time, the ability of an athlete to produce force rapidly should also be considered crucial. The results of the present study demonstrated that ratio scaling (i.e., relative to body mass) force at 150-, 200- and 250 ms and presenting absolute force at each of these time-points as a percentage of peak force resulted in no meaningful difference between female and male athletes. This not only highlights the importance of developing high relative strength in athletes, but when attempting to compare force production capabilities between women and men, ratio scaling data should be considered essential. However, researchers and practitioners should also be mindful that in some instances absolute force may be of primary importance (e.g., power lifting and weightlifting). The expression of peak force was found to explain 59%, 77% and 89% of the variance in net force produced at 150-, 200- and 250 ms, respectively, highlighting that the development of maximal force production should be considered essential to enhance early force production (<250 ms) in athletes. However, due to differences in muscle cross sectional area between males and females, it could be considered important for women athletes to build a stronger foundation via a greater focus on hypertrophic adaptations in the early phases of training to eventually realise higher maximal and rapid force producing characteristics. When developing resistance training programs for athletes’ practitioners should base the athletes needs on their relative force generating capacity.

## Supporting information

S1 Data (XLSX)
